# Primary stability of calcar-guided short-stem total hip arthroplasty in the treatment of osteonecrosis of the femoral head: migration analysis using EBRA-FCA

**DOI:** 10.1007/s00402-020-03610-4

**Published:** 2020-10-04

**Authors:** Yama Afghanyar, Christoph Danckwardt, Miriam Schwieger, Uwe Felmeden, Philipp Drees, Jens Dargel, Philipp Rehbein, Karl Philipp Kutzner

**Affiliations:** 1 Department of Orthopaedic Surgery, St. Josefs Hospital Wiesbaden, Beethovenstr. 20, 65189 Wiesbaden, Germany; 2 Department of Orthopaedics and Traumatology, University Medical Centre of the Johannes Gutenberg-University of Mainz, Langenbeckstraße 1, 55131 Mainz, Germany

**Keywords:** Osteonecrosis of the femoral head, Short stem arthroplasty, Total hip arthroplasty, Optimys, EBRA, Migration

## Abstract

**Introduction:**

Osteonecrosis of the femoral head (ONFH) is a disabling condition that often results in secondary arthritis necessitating total hip arthroplasty (THA). Short-stem THA has constantly gained popularity. It remains controversial, whether ONFH represents a risk factor for failure after the implantation of short stems with pronounced metaphyseal anchorage. The potential spread of the osteonecrotic area and bone marrow edema into the metaphyseal bone might result in compromised stability. Early implant migration is considered predictive of subsequent aseptic loosening. The purpose of this study was a migration analysis of a modern, calcar-guided short-stem implant in patients with ONFH in a mid-term follow-up.

**Materials and methods:**

This retrospective analysis investigated the migration pattern of 45 calcar-guided short stems in patients with ONFH, using Einzel-Bild-Roentgen-Analyse Femoral-Component-Analysis (EBRA-FCA). Influencing factors such as ARCO categories, age, gender, body weight and BMI were analyzed. Complications and adverse events were documented.

**Results:**

At mid-term [48.1 months (SD 20.7 months)], mean axial migration was 1.56 mm (SD 1.77 mm). Mean migration rate stabilized after 2 years. No influence of ARCO categories, age and BMI was found. A tendency of increased axial migration was observed in male patients and in overweight patients. No revision surgeries had to be performed during follow-up.

**Conclusion:**

The results indicate a migration pattern comparable to that of primary osteoarthritis patients with slight initial migration under full load followed by subsequent stabilization in the metaphyseal femur. The 100% survival rate at mid-term supports the usage of this short-stem design in patients with ONFH.

## Introduction

Osteonecrosis of the femoral head (ONFH) is a disabling condition that usually results in progressive femoral head collapse and end-stage secondary arthritis of the hip [1]. ONFH often affects young adults aged between 35 and 55 years [2]. Factors for the compromised blood supply of the femoral head, which is suspected to be the trigger of ONFH, include smoking, alcohol, various lipid metabolism disorders, corticosteroid therapy or neoadjuvant tumor therapies [2].

The treatment opportunities for early-stage ONFH are various and challenging [3, 4]. However, end-stage ONFH most likely makes total hip arthroplasty (THA) necessary [3, 5]. Given the commonly affected population of young patients, the ideal treatment option includes pain relief, allows returning to physical activities and best preserves femoral bone for future revision options [6].

Short-stem THA has become increasingly popular [7–11]. Short stems present as a bone and soft-tissue preserving alternative to conventional stems and offer the opportunity for revision with a standard-length stem if needed [12]. A great variety of short stems have been introduced to the market in the past decade, providing diverse philosophies and different types of anchoring [11]. Short stems of the newest generation cannot be easily classified, since they can be both calcar loading with pronounced metaphyseal anchorage, as well as diaphyseal anchoring, depending on the individual stem alignment [13, 14]. Thus, particularly in Europe, the term “calcar-guided” short stems have been established [15].

There are still uncertainties regarding the arthroplasty method that provides the most promising results in patients with ONFH. It remains controversial, whether ONFH represents a risk factor for failure after the implantation of short stems with pronounced metaphyseal anchorage [16–18]. The major concern in patients with ONFH is, that not only the femoral head but also the metaphyseal area is affected by osteonecrosis and potentially poor bone quality, subsequently being associated with an increased risk of migration and loosening.

Early implant migration is considered an indicator for subsequent aseptic loosening and mechanical failure [19]. Migration of more than 1.5 mm over the first 2 years postoperatively has been shown to be associated with an increased risk of revision in conventional cementless THA [19]. Another investigation found a threshold of 2.7 mm at 2 years for cementless stems being at risk for subsequent failure [20]. Measurements using “Einzel-Bild-Roentgen-Analyse—femoral component analysis (EBRA-FCA) resulted in a mean axial migration of 1.50 mm at mid-term in patients with primary and secondary osteoarthritis treated with a calcar-guided short stem [11]. Most of the stems stabilized over time. At mid-term no stem revision had to be performed [11]. To date, the migration pattern of any short-stem design has not been investigated in patients with ONFH.

This study aimed to investigate the migration pattern of a bone preserving and calcar-guided short-stem in patients with particularly the diagnosis of ONFH, using EBRA-FCA. The authors hypothesized that the investigated short-stem design can safely be used in patients with ONFH and that the migration pattern does not differ compared with the findings, previously published in patients with primary osteoarthritis [7, 10, 11, 21, 22].

## Material and methods

In the present investigation, 45 hips in 40 patients were included retrospectively after ethical approval (FF 104/216) as part of an ongoing observational study. This study encompassed patients with advanced ONFH who underwent short-stem THA in our department between 2011 and 2015. Prior to inclusion, written and verbal permission to participate has been obtained from all patients.

The inclusion criteria were age over 18 years, underlying diagnosis of ONFH with ARCO stage III or IV and sufficient bone quality classified as Dorr Type A or B. In most cases, a preoperative MRI was performed in the process of diagnosing ONFH.

Out of the 45 hips included, six hips were classified ARCO III and 39 hips were classified ARCO IV. 30 hips were classified Dorr type A and 15 hips were classified Dorr type B.

In the majority of cases, no specific risk factors for the development of ONFH could be identified (26 patients, 65%). Five patients reported a previous fracture of the femoral neck (12.5%). Three patients reported medication of corticosteroids due to various diseases (7.5%). Nicotine abuse was reported in 3 patients (7.5%). Of the remaining patients, one each presented with diabetes, alcohol abuse and an epiphysiolysis capitis femoris in the history.

Surgery was performed in 22 women and 23 men with a mean age of 61.1 years (SD 13.5; range 19.5–80.5). Five patients were treated with bilateral simultaneously. The mean body mass index was 28.1 kg/m^2^ (SD 5.1 kg/m^2^) (Table 1).

**Table 1:** Details of patients

Parameters	Results
Number of hips (*n*)	45
Gender (male/female)	23/22
Mean age (years) (range)	61.1 (19.5–80.5)
Mean BMI (kg/m^2^) (SD)	28.1 (5.1)
Mean weight (kg) (SD)	86.0 (21.0)
Mean height (m) (SD)	174.2 (11.2)
Etiology of ONFH (*n*) (%)	
Posttraumatic	5 (12.5)
Corticosteroid	3 (7.5)
Nicotine abuse	3 (7.5)
Alcohol abuse	1 (2.5)
Diabetes mellitus	1 (2.5)
Epiphysiolysis capitis femoris	1 (2.5)
Underlying condition unknown	26 (65)
ARCO classification (*n*) (%)	
ARCO III	6 (13.3)
ARCO IV	39 (86.7)
Dorr type (*n*) (%)	
Dorr A	30 (66.7)
Dorr B	15 (33.3)

**Fig. 1:**
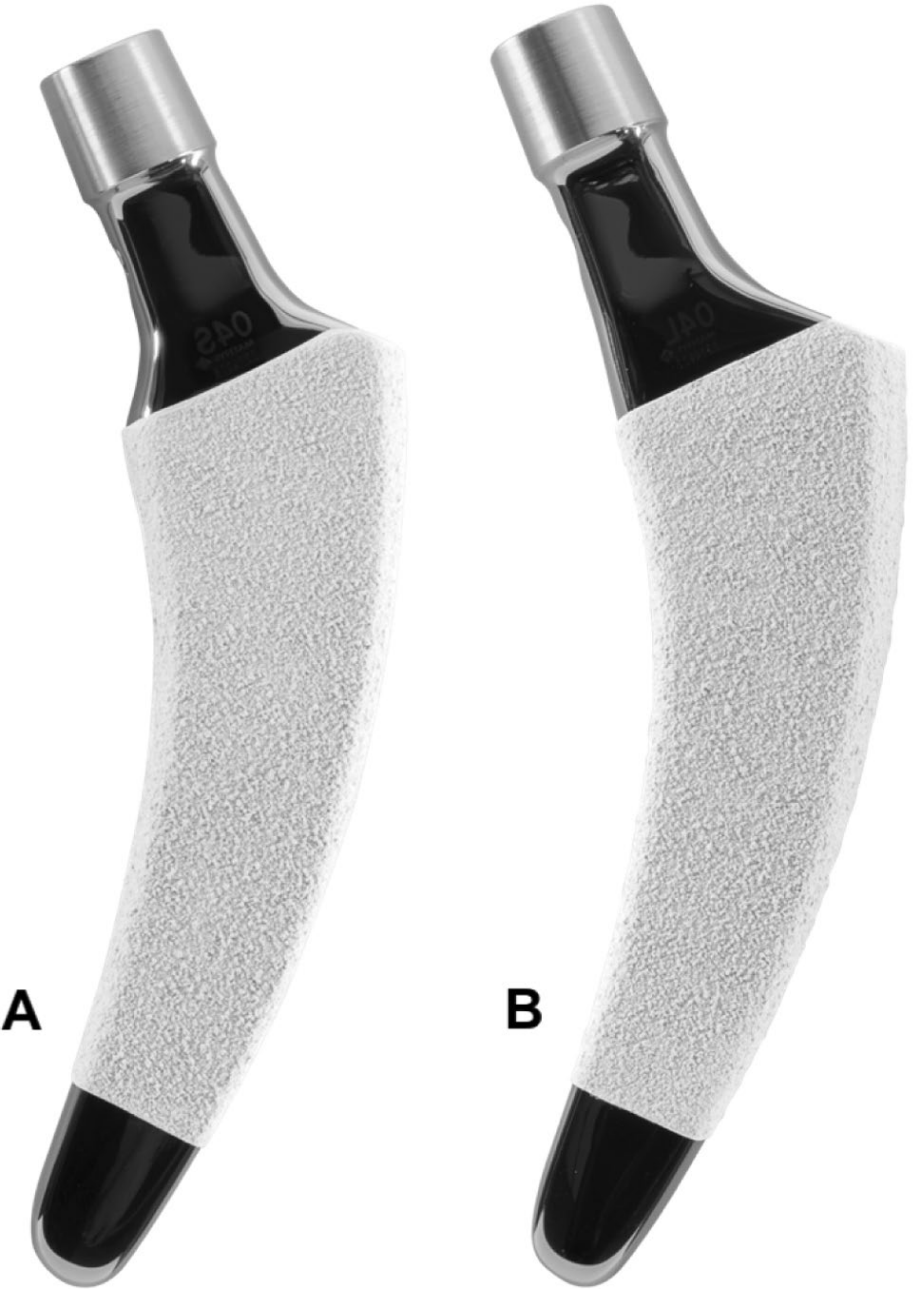
The optimys stem (Mathys Ltd., Bettlach, Switzerland) in two different offset versions (**a** standard; **b** lateral)

In all cases, the calcar-guided short stem optimys (Mathys Ltd., Bettlach, Switzerland) was implanted (Fig. 1). This stem can be classified as a type 2 B short-stem according to the classification of Khanuja et al. [23]. It is a femoral neck preserving prosthesis made of titanium alloy. The philosophy of the design is a pronounced bone contact at the calcar and at the distal lateral cortex. Thus, three-point anchoring is aimed for, but in some cases also a fit and fill in the proximal diaphysis is possible. The rough titanium plasma surface supports secure anchorage in the bone. Additionally, the stem contains an overlying calcium phosphate coating to enhance rapid osteointegration and achieve secondary stability [24]. To reproduce individual anatomy, two offset options are available.

The short stem was combined with cementless press-fit cups (*n* = 35 Fitmore, Zimmer Biomet GmbH, Winterthur, Switzerland; *n* = 10 RM Pressfit vitamys, Mathys Ltd., Bettlach, Switzerland) with a ceramic on polyethylene bearing couple. All surgeries were performed using a minimally invasive, antero-lateral approach in standardized surgical technique [25].

The operations were performed by experienced consultant surgeons. All patients were allowed full weight-bearing ambulation and started physiotherapy on the first day postoperatively.

Mean follow-up was 48.1 months (SD 20.7). The patients were followed up at 6 weeks postoperatively, at 12 months, at 24 months, and at 5 years, respectively.

Complications and adverse events during surgery and during follow-up were documented.

All patients underwent digital anteroposterior radiographs of the pelvis using a standardized technique. A positioning splint with 20° internal rotation of the hip joint was used [24].

Stem migration was analyzed using the “Einzel-Bild-Roentgen-Analysis Femoral Component Analysis” (EBRA-FCA) software (Institute for Basic Engineering Sciences, University of Innsbruck, Austria) [26]. The methodology was originally developed by Krismer et al. [27]. The EBRA-FCA software detects axial migration and tilt in the frontal plane [26]. Images are calibrated with the diameter of the implant head. Overall 19 reference points are defined on the femoral head (7), the stem (2), the femoral cortex (8), and one at the greater and lesser trochanter [26]. These reference points define predetermined distances, which are compared by the EBRA-FCA software to calculate implant migration. For the EBRA-FCA measurements, a series of at least three radiographs was needed. Radiographs with significant positioning artefacts were excluded by the software.

Patients were assigned into groups to evaluate patient-related factors on axial stem migration. Groups were divided according to ARCO stages (ARCO III vs. ARCO IV), age (< 65 years vs. > 65 years), gender (male vs. female), bodyweight (< 80 kg vs. > 80 kg) and BMI (< 30 kg/m^2^ vs. > 30 kg/m^2^) (Table 2).

**Table 2:** Influencing factors on axial migration

	Hips (*n*)	Mean migration (mm)	SD (mm)	Median migration (mm)	Range (mm)	Wilcoxon-test (*p*)
Gender	45	1.56	1.77	1.38	− 1.62 to 6.69	0.08
Male	23	2.11	1.92	1.39	− 0.47 to 6.69	
Female	22	0.98	1.42	1.14	− 1.62 to 3.22	
ARCO categories						0.15
III	6	2.78	2.46	2.23	0.22 to 6.69	
IV	39	1.37	1.60	1.18	− 1.62 to 5.15	
Age categories						0.31
< 65 years	24	1.36	1.97	1.16	− 1.62 to 6.69	
> 65 years	21	1.78	1.53	1.39	− 0.76 to 5.04	
Weight categories						0.18
< 80 kg	24	1.15	1.61	1.16	− 1.62 to 5.04	
> 80 kg	21	2.03	1.87	1.55	− 0.22 to 6.69	
BMI categories						0.65
< 30 kg/m^2^	31	1.39	1.63	1.38	− 1.62 to 5.04	
> 30 kg/m^2^	14	1.94	2.06	1.35	− 0.22 to 6.69	

### Statistical analysis

**Fig. 2:**
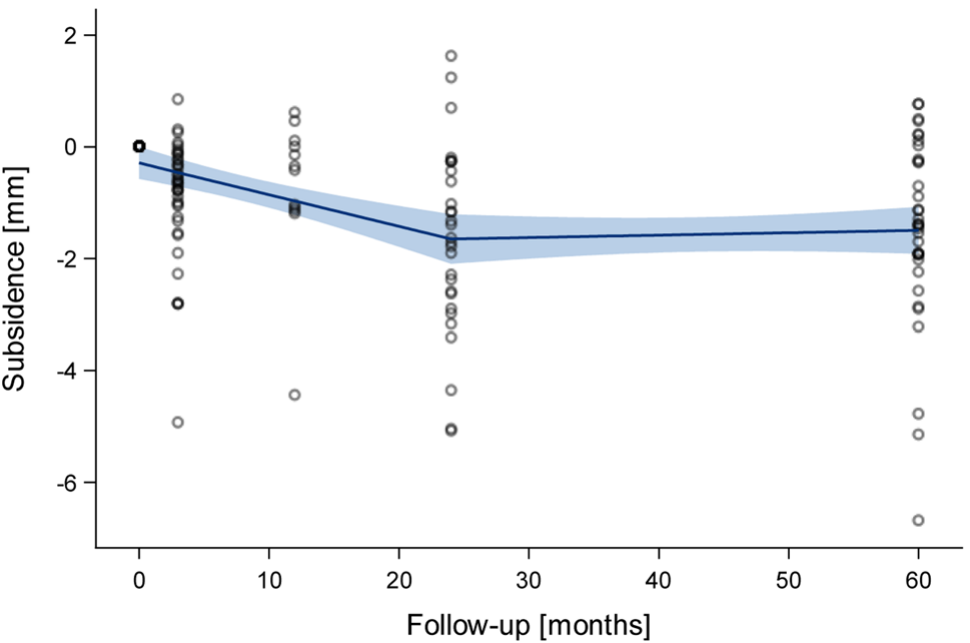
LOESS fitting of mean axial migration during follow-up

All analyses were performed using standard descriptive statistics such as mean, standard deviation (SD) and median (range). For statistical evaluation of stem tilt and subsidence, respectively, the last follow-up record was used. Differences were examined non-parametrically using Wilcoxon-two-sample-tests. For statistical significance, a *p *value of less than 0.05 was considered. As a complement for the visual display of the time-course of mean axial migration (Fig. 2), a locally weighted polynomial regression model based on the LOESS method was applied. All statistical analyses were performed using SAS Vs. 9.4 (SAS Institute Inc., Cary, USA).

## Results

At mid-term, 45 hips out of 40 patients contributed to the EBRA-FCA analysis. A total of 159 radiographs were available for the measurements.

The mean axial migration at mid-term was 1.56 mm (SD 1.77 mm). The median value was 1.38 mm (range − 1.62–6.69 mm) which is considerably lower than the mean value reflecting both skewed distribution and some outliers. After 2 years, a stabilisation was found (Fig. 2).

Axial migration of more than 1.5 mm at last follow-up was detected in 20 hips (44.4%).

There were no statistically significant differences between the ARCO stages. Hips classified with ARCO III showed a mean axial migration of 2.78 mm (SD 2.46 mm) and hips classified ARCO IV showed a mean migration of 1.37 mm (SD 1.60 mm) (*p* = 0.15). In addition, no significant differences were found in the different age groups (*p* = 0.43) and in the different BMI groups (*p* = 0.65) (Table 2).

In contrast, a tendency of increased axial migration in men compared to women was found (*p* = 0.08). At last, follow-up mean axial subsidence was 2.11 mm (SD 1.92 mm) in the male group and 0.98 mm (SD 1.42 mm) in the female group.

Comparing weight groups of < 80 kg versus > 80 kg, subsidence was measured to be 1.15 mm (SD 1.61 mm) versus 2.03 mm (SD 1.87 mm), respectively (*p* = 0.18). The trend suggests that heavy body weight in male patients is associated with enhanced axial migration (Table 2).

**Fig. 3:**
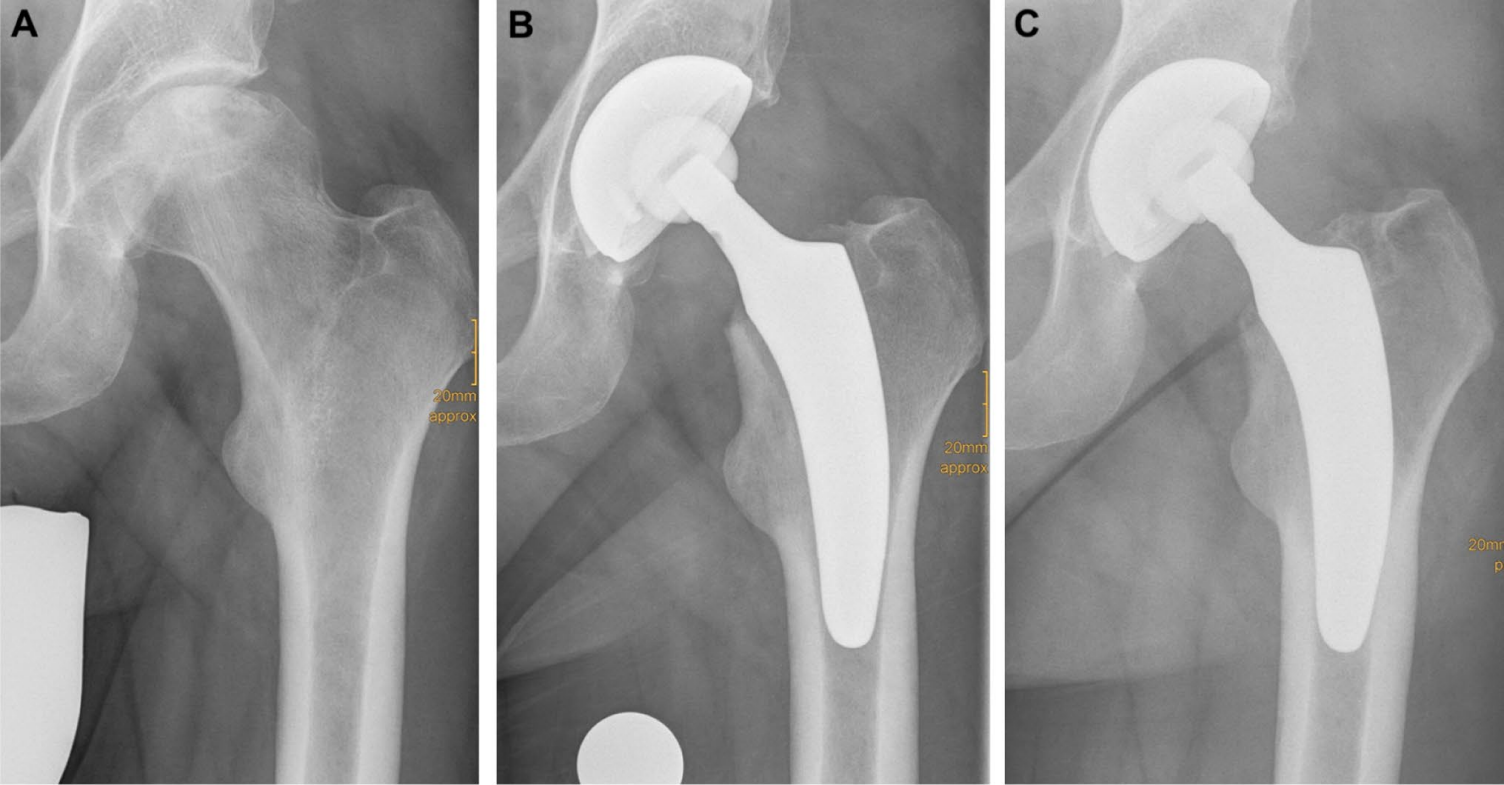
Radiographs of a 49-year-old male patient (weight: 110; height: 186 cm) with ONFH. **a** Preoperative (ARCO stage IV and Dorr type A); **b** postoperative; **c** mid-term follow-up (axial migration of 5 mm, without signs of stem loosening)

Two patients presented outlier results. One patient was treated bilaterally and showed axial migration of > 5 mm on both sides (left: 5.15 mm; right: 6.69 mm). Another patient also showed pronounced migration of 5.04 mm (Fig. 3). It was noticeable that both outliers were male and presented with heavy bodyweight.

Mean stem tilt at mid-term follow-up was 0.53° (SD 2.41°). There was no evidence for significant differences between the different groups.

To date, there were no adverse events and no revision surgery was needed.

## Discussion

The present study aimed to analyze the migration pattern of a calcar-guided short stem using EBRA-FCA in patients with ONFH at mid-term. To date, no stem-related complications could be observed and none of the investigated implants required revision surgery. At mid-term, the mean axial migration resulted in 1.56 mm. In most cases, after 2 years a stabilization was observed. Subsidence of more than 1.5 mm at last follow-up was detected in 20 hips (44.4%). While no influence of age, BMI and ARCO classification was found, a tendency of increased axial migration was observed in male and heavy-weight patients.

The treatment of ONFH is challenging. It remains controversial, whether ONFH represents a risk factor for failure after the implantation of short stems. The main concern of using short-stem THA in patients with ONFH arises due to the potentially reduced bone quality and the osteonecrotic area beyond the femoral head also affecting the femoral neck and the metaphyseal bone. According to the critics, a THA designed for metaphyseal anchoring may be associated with poor primary stability, impaired osteointegration and thus an increased risk of loosening. Previous histological studies suggested that ONFH includes structional alterations not only of the femoral head but also the femoral neck [9, 28]. Tingart et al. analyzed the bone matrix composition and trabecular microarchitecture of the femoral metaphysis in patients with ONFH [29]. They concluded that alterations in bone metabolism and architecture might contribute to the higher rates of stem loosening after THA in patients with ONFH [28, 29]. Thus, to date, conventional THA with pronounced diaphyseal anchorage is considered the gold standard in patients with ONFH [2, 4, 5, 16]. Kim et al. [30] investigated the outcome of a conventional THA with a modular femoral component in patients with ONFH and younger than 50 years. The survival rate with the endpoint of stem revision for any reason was 93.8% and 100% for aseptic loosening at 16.8 years [30]. Garino et al. [31] reported a 96% survival rate in 123 cemented and hybrid THAs in patients with ONFH after 55 months.

There are only a few previous studies investigating short-stem THA in patients with ONFH. The newest generation of short stems aims at a physiological metaphyseal fixation and load transmission to reduce stress-shielding and to preserve the proximal femoral bone [32]. One of the most popular short stems, solely allowing metaphyseal anchorage, is the Metha stem (B. Braun, Tuttlingen, Germany), for which controversial outcomes in patients with ONFH have been published [16, 33]. Floerkemeier et al. [28] reported encouraging results using the Metha stem in a total of 73 hips in a mean follow-up of 34 months with only two revisions needed. Recently, Suksathien et al. [18] reported a Kaplan–Meier survivorship, with the endpoint being any stem revision, of 98.7% at 7 years. However, Schnurr et al. [33] compared 231 implantations of the Metha stem in patients with ONFH to 1455 operations in patients with primary osteoarthritis using data over a 10-year period. Whereas the total revision rate turned out not to be significantly increased in patients with ONFH compared to patients with primary osteoarthritis, however, they found that the aseptic loosening rate of the short stems was significantly elevated in those patients with ONFH. Particularly male patients and patients providing risk factors such as alcohol abuse, cortisol intake and radiation were prone to early revision surgery.

Very little data is available on primary stability and migration regarding short stems in patients with ONFH. Zeh et al. [9] concluded in a study using the Mayo stem (Zimmer Inc., Warsaw, USA) that no significant migration and tilt occurred in patients with ONFH after 7.9 years. However, they found a mean axial migration of over 3 mm and the method used has not been validated before.

Impaired stability and pronounced migration are considered an indicator for subsequent aseptic loosening and mechanical failure [19]. Krismer et al. [19] reported that axial migration of more than 1.5 mm after 2 years in conventional cementless THA was predictive for late aseptic loosening and a potential increase in the risk of revision. But it is still unknown if this prediction can be transferred to short-stem THA as well. Previous studies have performed migration analyses in short-stem THA following the indication of primary osteoarthritis using EBRA-FCA [7, 21, 34]. Kutzner et al. [34] investigated the optimys stem in patients with primary and secondary osteoarthritis. Axial migration of 1.43 mm at 2 years was reported. 39.6% of the stems showed subsidence of 1.5 mm or more [34]. However, at mid-term, no significant further migration was observed. In only four hips, due to undersizing as part of a surgical mistake, stems did not stabilize after 2 years. At mid-term, however, no stem revision was needed [11]. Another study, analyzing the Fitmore stem (Zimmer Inc., Warsaw, USA) reported a mean axial migration of 1 mm after 2 years. A potential critical migration of more than 1.5 mm was detected in 25% of the investigated hips [21]. Again, at mid-term, all stems stabilized. No implant failure was observed, neither in the group of implants with early stabilization, nor the group with extensive early-onset migration [10]. These findings are in line with previous publications regarding different stem designs. Floerkemeier et al. [35] in a prospective radiostereometric analysis (RSA) study using the Metha stem, found increased early migration, but again not being associated with a higher risk of subsequent implant failure. A migration analysis of the Nanos stem (Smith and Nephew GmbH, Marl, Germany) also confirmed slight initial migration within three months after surgery, followed by secondary stabilisation, suggesting a low risk of aseptic loosening [36]. Only one stem revision due to postoperative periprosthetic fracture was observed.

To date, to our best knowledge, no analysis of the migration pattern of a new-generation short-stem design in patients with ONFH has been published, using a validated method like EBRA-FCA. The results of the present study showed similar outcomes compared to the previously published data in patients with osteoarthritis [7, 10, 21, 34]. As it was found in patients with osteoarthritis, at mid-term no stem failure and revision occurred. These findings strongly support that the optimys stem is a safe option in the treatment of patients with ONFH. They indicate a sufficient primary stability and successful osteointegration also for this group of patients. Both, the results in patients with osteoarthritis as well as the outcomes of the present study suggest that the 1.5 mm threshold of axial migration may not be valid for predicting aseptic loosening and implant failure in calcar-guided short-stem THA. However, further monitoring of those stems with pronounced initial subsidence is obligatory to detect potential signs of loosening and failure.

In accordance with the previously published data on patient-related factors, which influence the rate of stem migration of the investigated stem design, again, male and heavy-weight patients are to be considered at risk for pronounced early migration [34]. The present mid-term results in patients with ONFH confirm these findings. This is in line with previously published data using different stem designs [21, 37]. However, in a retrospective analysis of migration data from two different short stem studies using the Metha stem and the Nanos stem, factors, including age, height, weight and gender, did not affect the migration pattern [38]. It seems obvious that migration patterns of different stem designs, providing different concepts of anchorage, may be affected differently by patient-related influencing factors.

Besides patient-related factors, surgical technique highly influences stabilization into the femoral bone, especially in heavy-weight patients. Stems providing a poor fit-and-fill into the bone and lack of cortical contact have been reported to show reduced primary stability [11]. Surgeons, therefore, are highly recommended to use intraoperative radiography to confirm correct positioning and sizing intraoperatively [39].

**Fig. 4:**
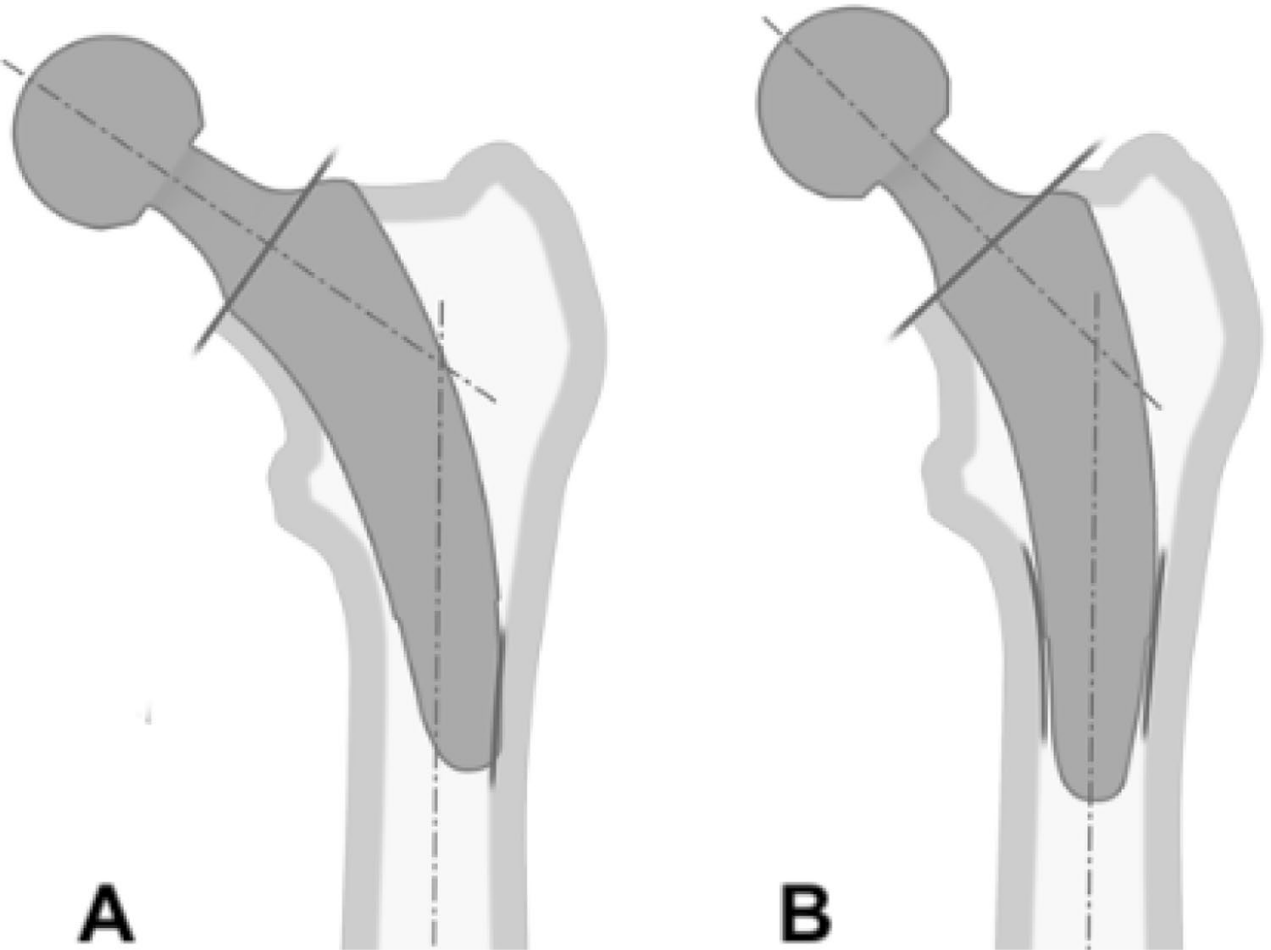
Depending on the spread of the area of ONFH, the stem alignment can be done individually. **a** Three-point fixation with metaphyseal anchorage; **b** additional fit-and-fill fixation in the proximal diaphysis

The design of calcar-guided stems, such as the optimys stem, differs to that of the early short stem designs with solely metaphyseal anchorage, such as the Metha stem. Whereas most varus hips achieve stabilization by three-point fixation in the metaphyseal bone, in calcar-guided short-stem THA, due to the design properties, some neutral and most valgus hips may also be stabilized by supplementing an additional fit-and-fill fixation in the proximal diaphysis [7] (Fig. 4). Already in 2012, Floerkemeier et al. [28] found in a review of short- to mid-term results of short stems in patients with ONFH predominantly good outcomes. However, marked differences in the desgin of short stems and their type of anchorage had to be acknowledged. They concluded, that those short stems with primary or additional diaphyseal fixation do not reveal an increased risk of failed osseointegration or loosening. For designs with a primary metaphyseal anchorage, and the osteonecrosis exceeding the femoral neck, an implantation could not be recommended. Regarding the successful achievement of sufficient primary stability, especially in hips with ONFH, the design properties of calcar-guided short stems, given an individualized meta-diaphyseal anchorage, may therefore account for significant advantages compared with earlier short-stem designs. This can be confirmed by previously published data. Jerosch et al. published mid-term results of the calcar-guided MiniHip stem (Corin Medical, Cirencester, UK) with 100% stem survival and encouraging clinical outcome [17]. Furthermore, Capone et al. found excellent clinical results of the calcar-guided Nanos stem (Smith and Nephew, Marl, Germany) and successful osteointegration at mid-term without any revision needed for any reason [40].

**Fig. 5:**
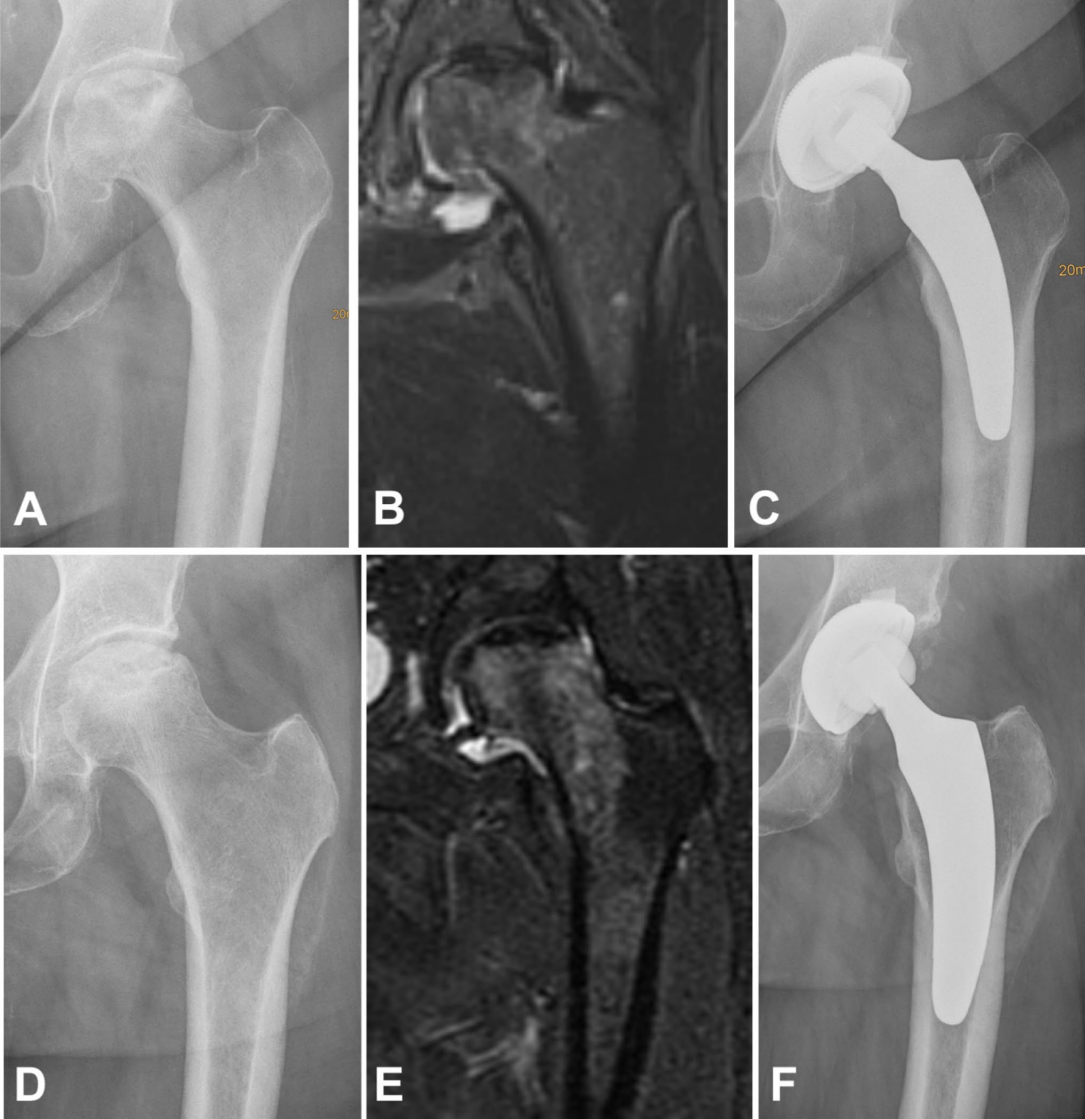
Upper row: if only affecting the femoral head, metaphyseal anchoring based on three-point-anchoring can be aimed for (**a** preoperative radiograph; **b** MRI; **c** postoperative radiograph). Lower row: if also affecting the femoral neck and large parts of the metaphysis, an additional pronounced diaphyseal anchorage should be pursued (**d** preoperative radiograph, **e** MRI; **f** postoperative radiograph)

For the safe usage of calcar-guided short stems, a preoperative MRI may be helpful. Depending on the spread of the area of ONFH, the stem alignment can be done individually. If only affecting the femoral head, metaphyseal anchoring based on three-point-anchoring can be aimed for. If also affecting the femoral neck and large parts of the metaphysis, an additional diaphyseal anchorage should be pursued (Fig. 5).

The present study has several limitations. First, the mid-term follow-up does not allow definite conclusions about the long-term outcome of short-stem THA in patients with ONFH. However, early migration analysis may allow a prediction of implant survival and may indicate undesirable results. Second, MRI has not been carried out on every patient to identify the precise amount of osteonecrosis. In those hips with radiological documented already fractured subchondral femoral bone, an ARCO stage IV was assumed. Thus, a proof of metaphyseal involvement has not been supplied. Third, the fact that a control group is missing in the study design does not allow for a direct comparison of patients with ONFH and primary osteoarthritis. However, data on patients with osteoarthritis has previously been published by the same study group, using the same implants and the identical standardized postoperative care. Another limitation results in the EBRA software failing to evaluate all radiographs. The requirements for EBRA measurement are quite challenging, leading to a some of the radiographs not being accepted by the EBRA software. Furthermore, radiostereometric analysis (RSA) provides higher accuracy in comparison to the EBRA method. The accuracy of EBRA-FCA has been reported to be ± 1 mm for subsidence, with a specificity of 100% and a sensitivity of 78% to detect migration [27]. RSA, however, requires the implantation of markers intraoperatively and would have caused intense cost and effort.

## Conclusion

The optimys stem is a safe option in the treatment of patients with ONFH. The results indicate a migration pattern comparable to those previously published in patients with primary osteoarthritis. Initial migration under full load is followed by subsequent stabilization in the metaphyseal femur. Male and heavy-weight patients showed an increased initial migration. The survival rate of 100% at mid-term is remarkable. The design properties of calcar-guided short stems, along with the individual meta-diaphyseal anchorage, may account for significant advantages in patients with ONFH compared with earlier, solely metaphyseal anchoring, short-stem designs. Long-term studies are obligatory.

## Data Availability

The dataset generated and/or analysed during the current study are not publicly available due to the high volume of data but are available from the corresponding author on reasonable request..
